# It's Like We Speak a Different Language: Support Needs and Preferences of Older LGB Women Who Have Lost a Spouse or Partner

**DOI:** 10.1093/geroni/igaa057.221

**Published:** 2020-12-16

**Authors:** Korijna Valenti

**Affiliations:** Miami University, Oxford, Ohio, United States

## Abstract

Needs during and following end-of-life (EOL) experiences are distinctive for sexual and gender minority (SGM) people and, in particular, older lesbian, gay, and bisexual women (LGB) women; however, access to supportive services is limited. This poster presents findings from a qualitative study of older (60+) LGB women who have lost a spouse or partner. This work draws on queer gerontological theory, addressing issues of individual agency, systemic silence, and marginalization by invisibility of older SGM women in order to illuminate specific areas in which policy may be changed and improved. The study analyzed participants’ (n = 16) reflections on their experiences of losing a loved one, including how they sought out and received essential grief support and the type of support they would have preferred and from whom, particularly immediately following their spouse or partner’s death. Thematic analysis revealed three main findings: 1) having a women-identifying support presence at the time of death for both themselves and their spouse or partner; 2) needing LGB women (or women allies) during EOL for support; and 3) preferring grief groups comprised of other LGB women (or women allies) based on their feelings of difference from gay men and heterosexual/non-SGM women and men. Findings reveal the need for a better understanding among healthcare and social work support professionals regarding LGB women’s grief needs and preferences; grief options (e.g. lesbian and non-monosexual (bi+) grief groups); and how to implement policy changes reflecting these needs and preferences.

